# Antiproliferative Properties of Newly Synthesized 19-Nortestosterone Analogs Without Substantial Androgenic Activity

**DOI:** 10.3389/fphar.2018.00825

**Published:** 2018-07-27

**Authors:** András Gyovai, Renáta Minorics, Anita Kiss, Erzsébet Mernyák, Gyula Schneider, András Szekeres, Erika Kerekes, Imre Ocsovszki, István Zupkó

**Affiliations:** ^1^Department of Pharmacodynamics and Biopharmacy, University of Szeged, Szeged, Hungary; ^2^Department of Organic Chemistry, University of Szeged, Szeged, Hungary; ^3^Department of Microbiology, University of Szeged, Szeged, Hungary; ^4^Department of Biochemistry, University of Szeged, Szeged, Hungary; ^5^Interdisciplinary Centre for Natural Products, University of Szeged, Szeged, Hungary

**Keywords:** 19-nortestosterone analogs, antiproliferative action, HeLa Cells, tubulin polymerization, androgenic activity, cell cycle, caspase

## Abstract

19-Nortestosterone C-17 epimers with prominent antiproliferative properties have been previously described. In our present study, five novel 17α-19-nortestosterones (**3−7**) were synthesized to increase their beneficial biological activities with no associated undesired hormonal effects. The compounds were screened by a viability assay against a panel of human adherent gynecological cancer cell lines. Three of the tested derivatives (**3**−**5**) exhibited a remarkable inhibitory effect on the proliferation of HeLa cells with IC_50_ values lower than that of our reference agent cisplatin (CIS). These three active agents also displayed considerable cancer selectivity as evidenced by their weaker growth inhibitory effect on non-cancerous fibroblast cells compared to CIS. The most potent newly synthesized 17α-chloro derivative (**3**) was selected for additional experiments in order to characterize its mechanism of action. Since nandrolone (19-nortestosterone, **1**) is a structural analog with selective antiproliferative action on cervical carcinoma cells, it was utilized as a positive control in these studies. A lactate dehydrogenase (LDH) assay demonstrated a moderate cytotoxic effect of the test compounds. Cell cycle disturbance and the elevation of the hypodiploid population elicited by the test agents were detected by flow cytometry following propidium staining. The proapoptotic effects of the tested steroids were confirmed by fluorescent microscopy and a caspase-3 activity assay. Treatment-related caspase-9 activation without a substantial change in caspase-8 activity indicates the induction of the intrinsic apoptotic pathway. The selected agents directly influence the rate of tubulin assembly as evidenced by a polymerization assay. Yeast-based reporter gene assay revealed that the androgenic activity of the novel 19-nortestosterone derivative **3** is by multiple orders of magnitude weaker than that of the reference agent **1**. Based on the behavior of the examined compounds it can be concluded that a halogen substitution of the 19-nortestosterone scaffold at the 17α position may produce compounds with unique biological activities. The results of the present study support that structurally modified steroids with negligible hormonal activity are a promising basis for the research and development of novel anticancer agents.

## Introduction

Cancer is the second leading cause of death globally: in 2015 malignancies were responsible for 8.7 million deaths, and 17.5 million new cancer cases were registered worldwide. Based on incidence estimates the number of new cases is expected to rise by about 70% over the next two decades (Fitzmaurice et al., 2017). Besides numerous preventive strategies and early diagnosis, the research for and development of innovative anticancer agents is one of the most important approaches to decrease global cancer burden.

Steroidal agents used in oncological practice are typically administered for their endocrine disruptor properties (e.g., estrogen antagonists, aromatase inhibitors). Synthetic analogs of naturally occurring steroids are widely utilized in the treatment of cancers of the reproductive system (Lin et al., 2010; Sharifi et al., 2010).

Besides the well-known endocrine disruptors several other steroids have been reported to exert pronounced anticancer effects in a hormone-independent manner. 2-Methoxyestradiol, an endogenous metabolite of estradiol without hormonal activity, exhibits a potent antiproliferative action against various tumor cell lines *in vitro*, and inhibits tumor growth *in vivo* (Fotsis et al., 1994). It is also demonstrated to induce programmed cell death in endothelial cells and suppresses cancer-related angiogenesis (Yue et al., 1997; LaVallee et al., 2002).

Cardiac glycosides are a group of steroidal compounds traditionally utilized in the management of congestive heart failure. Epidemiological studies have revealed that many of them, including digitoxin, oleandrin, bufalin, and calotropin exert a potent anticancer effect against different malignancies via the inhibition of proliferation and apoptosis induction involving complex cell signal transduction mechanisms (Mijatovic et al., 2007; Newman et al., 2008).

Steroidal alkaloids are nitrogen containing secondary metabolites found in many plant families (e.g., Liliaceae, Solanaceae), and many of them are well characterized as potent anticancer agents against human malignant cell lines (Koduru et al., 2007). Solasodine glycosides have been investigated in the clinical setting against basal cell carcinoma, and a locally applied cream was found to be effective in a substantial proportion of patients (Punjabi et al., 2008).

Androstanes and their structural analogs are regarded as a promising skeleton for the development of steroid-based anticancer agents. A large body of evidence indicates the outstanding importance of these compounds and their versatile antitumor effects. A considerable antiproliferative action of several sets of innovative androstane analogs have been reported against a broad variety of cell lines, including prostate, breast, cervix, ovarium, leukemia, melanoma, colon, and gastric cancers (Iványi et al., 2012; Ajdukovic et al., 2013, 2015; Acharya and Bansal, 2014; Cui et al., 2015; Jakimov et al., 2015). 19-Nortestosterone derivatives, e.g., levonorgestrel, desogestrel, and dienogest, an important division of testosterone-derived molecules are widely utilized in hormone replacement therapy (Campagnoli et al., 2005), contraception (Minami et al., 2013; Royer and Jones, 2014), and treatment of endometriosis (Minami et al., 2013; Miyashita et al., 2014). Beyond these well-established clinical applications, several 19-nortestosterone derivatives have recently been reported as potential anticancer agents. Mibolerone (7α,17α-dimethyl-19-nortestosterone), a metabolically stable synthetic member of this class has been demonstrated to effectively inhibit estrogen-stimulated breast cancer cell proliferation *in vitro* (Cops et al., 2008).

Tibolone, a selective regulator of tissue estrogen activity for postmenopausal women, is also known to induce apoptosis in breast cancer cells *in vitro*, and has been demonstrated to suppress tumor growth in animal models (Franke and Vermes, 2003; Erel et al., 2006). Further, 19-nortestosterones, such as gestodene and 3-ketodesogestrel exhibit antitumor activity against several breast cancer cell lines *in vitro*, as well as *in vivo*, in rat model of breast cancer (Kloosterboer et al., 1994). Additional, 19-nortestosterone derivatives as potential proliferation inhibitors in brain, prostate, and renal cancer cell lines have also been described (Mohamed et al., 2015).

Although several analogs truly possess a promising anticancer effect, their actions are mainly mediated by their hormonal activity, hindering a wide-scale utilization of these compounds in cancer therapy.

Since the 17β-hydroxy function of endogen androgens play a crucial role in the molecule’s interaction with its hormone receptors, modifications of this group reduce hormonal activity. The lack of the C-19 methyl group also decreases the hormonal properties of such analogs substantially (Fragkaki et al., 2009). In a previous research, we have reported on the synthesis of a series of 17-substituted 19-nortestosterone derivatives and demonstrated their antiproliferative properties against human ovarium, cervix, and breast cancer cell lines. Nandrolone (19-nortestosterone, **1**) was found to exhibit a selective proliferation inhibitory effect against cervical cancer cells (HeLa) at low concentrations (Schneider et al., 2016). As an extension of this previous research a set of novel 19-nortestosterone analogs with various substituents at position C-17 have been synthesized. Recent reports about halogen-substituted androstane-derivatives with an increased *in vitro* anticancer activity encouraged us to introduce halogens in order to enhance the antiproliferative activity (Banday et al., 2010; Iványi et al., 2012). The aims of our current study were to assess the antiproliferative properties of these analogs, including tumor selectivity, as well as to characterize the mechanism of action of the most potent compound. Since, the endocrine actions of steroid-based drug candidates are exceptionally relevant, the androgenic potentials of the compounds were also tested.

## Materials and Methods

### Synthesis and Chemicals

The exact conditions applied for the preparation processes of the synthesized 19-nortestosterone analogs **2−7** and their detailed characterization are provided as Supplementary Material. 10 mM stock solutions of the tested agents were prepared with dimethyl sulfoxide (DMSO) for all *in vitro* experiments. The medium with the highest DMSO concentration (0.3%) did not exert any notable effect on cell proliferation. Unless otherwise specified, all other chemicals and kits were purchased from Sigma-Aldrich Ltd. (Budapest, Hungary).

### Cell Cultures

Gynecological cancer cell lines, including ovarian (A2780), cervical (HeLa), and breast cancer cell lines (MCF7, T47D, MDA-MB-231, and MDA-MB-361) were purchased from the European Collection of Authenticated Cell Cultures (ECACC, Salisbury, United Kingdom). Additional cervical cell lines (SiHa and C33A) and a non-cancerous immortalized, mammary gland epithelial cell line (hTERT-HME1) were purchased from LGC Standards GmbH (Wesel, Germany). Non-cancerous fibroblast cells (MRC-5) were also obtained from the ECACC. All cells were cultured in minimal essential medium supplemented with 10% fetal bovine serum, 1% non-essential amino acids, and 1% antibiotic-antimycotic mixture, in humidified air containing 5% CO_2_ at 37°C. Immortalized hTERT-HME1 cells were maintained in serum-free mammary epithelial cell growth medium (MEGM) supplemented with insulin, human epidermal growth factor (hEGF), hydrocortisone, bovine pituitary extract, and an antibiotic-antimycotic mixture. All the medium and supplements were purchased from Lonza Group Ltd. (Basel, Switzerland).

### Assessing the Antiproliferative Effect

The antiproliferative properties of the compounds were assessed by an MTT assay (Mosmann, 1983). Cells were seeded onto 96-well microplates at a density of 10,000 cells/well (MDA-MB-361 and C33A) or 5,000 cells/well (all other cell lines). After an overnight incubation, fresh medium containing the test compounds (at a concentration of 10 or 30 μM) was added. After incubation for 72 h at 37°C in humidified air, [3-(4,5-dimethylthiazol-2-yl)-2,5-diphenyltetrazolium bromide] (MTT) solution (5 mg/mL) was added. Purple formazan crystals were formed by the living cells during a 4 h contact period, which were assayed by spectrophotometry after having been dissolved in 100 μL DMSO. Untreated cells served as control, and cisplatin (CIS) (Ebewe Pharma GmbH, Unterach, Austria) was used as a reference compound. When a test agent elicited over 50% growth inhibition at the 30 μM concentration, the assay was repeated with a series of dilutions (0.1−30 μM) and IC_50_ values were calculated (GraphPad Prism 5.0, GraphPad Software, San Diego, CA, United States). Two independent measurements were performed with five parallel wells. To present preliminary data concerning tumor selectivity of the potent compounds, the procedure was repeated on MRC-5 fibroblast and hTERT-HME1 immortalized mammary gland epithelial cells under the same experimental conditions.

### Assessing the Cytotoxic Effect

The direct cytotoxic effects of the test agents were determined by a lactate dehydrogenase (LDH) assay. Cells were seeded onto 96-well microplates at a density of 5,000 cells/well and were incubated overnight, after which the medium containing the test compounds at proper concentrations was added. After incubation for 24 h, the activity of LDH released by the treated cells was determined by a commercially available colorimetric kit according to the manufacturer’s instructions (Hoffmann-La Roche Ltd., Basel, Switzerland). Untreated cells served as control, while detergent Triton X-100 and CIS were used as reference agents.

### Flow Cytometric Analysis of Cell Cycle and Apoptosis

The distribution of cells in different cell cycle phases (subG1, G1, S and G2/M) was analyzed via the measurement of cellular DNA content by flow cytometry. HeLa cells were seeded onto 6-well plates and allowed to stand for an overnight. The cells were treated with the selected compounds for 24, 48, or 72 h. Then cells were harvested, washed and fixed in ice cold 70% ethanol and stored at −20°C at least for an hour. Next, a DNA staining solution (containing distilled water, propidium-iodide, Triton-X100, sodium citrate, and ribonuclease-A) was added to each sample and stored in the dark at room temperature for an hour. Stained cells were analyzed by flow cytometry (Partec CyFlow, Partec GmbH, Munster, Germany) with at least 20,000 cells being evaluated for each analysis. Data processing was performed using the ModFit LT 3.3.11 software (Verity Software House, Topsham, ME, United States).

### Morphological Studies Using Fluorescent Microscopy

Fluorescent double staining was performed in order to detect apoptosis induction and morphological changes using fluorescent microscopy. HeLa cells were seeded into a 96-well plate at the density of 3,000Ȓ5,000 cells per well. After an overnight incubation, the cells were treated with various concentrations of the test compounds for 24 h. The treated cells were then incubated with a fluorescent staining solution (containing Hoechst 33258 and propidium iodide, 500 and 300 μg/mL, respectively) for an hour. After staining, the cells were analyzed using a fluorescent microscope (Nikon ECLIPSE 146 TS100, Nikon Instruments Europe, Amstelveen, Netherlands) equipped with appropriate optical filters. For all different conditions, at least six fields were recorded with an attached QCapture CCD camera. This way cells with an intact, apoptotic or necrotic morphology can be distinguished based on their different nuclear morphological appearance and distinct membrane integrity.

### Caspase Activity Measurements

In order to detect, whether the test compounds induce programmed cell death, the activity of caspase-3 was determined by a colorimetric assay. To elucidate the exact pathway of apoptosis, activities of caspase-8 and caspase-9 were additionally determined by colorimetric kits. In all cases approximately 12 million cells were treated with appropriate concentrations of the compounds for 24 or 72 h. After the treatment, the cells were scraped and the enzyme activities were determined by means of colorimetric assays mentioned above. All kits were purchased from Abnova Corp. (Taipei, Taiwan) and used in accordance with the instructions of the manufacturer.

### Tubulin Polymerization Assay

In order to determine the direct action of the test compounds on the microtubular system, an *in vitro* tubulin polymerization assay was performed using a commercially available kit (Cytoskeleton Inc., Denver, CO, United States) in accordance with the provider’s instructions. The assay reactions were performed on a pre-warmed (37°C), UV-transparent 96-well microplate. Ten microliters of the test solutions were placed on the wells supplemented with 2 mM MgCl_2_, 0.5 mM ethylene glycol tetraacetic acid (EGTA), 1 mM guanosine triphosphate (GTP) and 10.2% glycerol. Ten microliters of general tubulin buffer was used as untreated control, and paclitaxel (PAC) served as the reference compound. The polymerization reaction was initiated by adding 100 μL of 3.0 mg/mL tubulin in 80 mM PIPES, pH 6.9, to each sample. Absorbance of the samples was measured per minute, at 340 nm, using a 60-min kinetic measurement protocol. Each sample was prepared in two parallels. To characterize the process, polymerization curves were fitted to the measured data. The highest difference between the absorbances measured at two consecutive time points was regarded as *V*_max_ (Δabsorbance/min) for the tested compound. A clinically applied reference agent, PAC was used at a relatively high concentration (10 μM) as recommended by the manufacturer. This concentration is approximately 1,000-fold higher than the IC_50_ value of PAC on HeLa cells (Jordan et al., 1996). Since similarly high concentrations of the tested compounds were not possible to be applied because of the limited solubility of the substances in the recommended buffer, we used the highest concentrations reflecting the differences in the efficacies of the tested compounds.

### Assessing Hormonal Effect

An endocrine bioassay kit (Xenometrix AG, Allschwil, Switzerland) was used to test for a potential residual androgenic activity of the selected agents. Genetically modified yeast cells (*Saccharomyces cerevisiae*) containing the human androgenic receptor gene integrated into a yeast chromosome, as well as an expression plasmid with the sequences of both the androgen responsive element and a lacZ reporter gene were cultured in humidified air at 31°C with agitation for 2 days. The appropriate concentrations of the test compounds and a CPRG substrate solution (chlorophenol red-β-D-galactopyranoside) for β-galactosidase were added into a 96-well microplate according to the instructions of the manufacturer. Androgen agonistic and antagonistic properties of the test compounds were determined by a colorimetric assay. For the antagonistic measurements the medium was supplemented with 5α-dihydrotestosterone (DHT). Once the reporter gene is expressed, β-galactosidase is secreted into the medium, and converts the yellow CPRG substrate into a red product, which can be quantified at 570 nm. Quantities of this red product correlate with the liberation of β-galactosidase, which is increased when an agonistic effect is present, while it is decreased when the test compound exerts an antagonistic effect. For the agonistic and antagonistic assays, nandrolone (**1**) and flutamide were used as reference agents, respectively.

### Statistical Analysis

In all experiments, the statistical evaluation of the results was performed by one-way analysis of variance followed by the Dunnett posttest, using the GraphPad Prism 5 software (GraphPad Software; San Diego, CA, United States). Mean values and the SEM were calculated in all cases.

## Results

### Synthetic Studies

**FIGURE 1:**
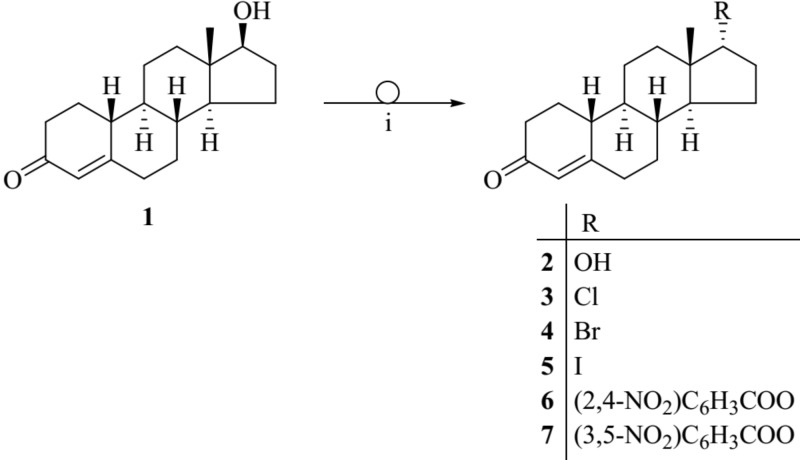
Chemical structures of the synthesized 19-nortestosterone derivatives. Applied reagents and conditions: (i) Ph_3_P, aryl acids or alkyl halogenides, diethyl azodicarboxylate, toluene, 80°C.

The Mitsunobu reaction is widely employed for the inversion of stereogenic centers of secondary alcohols including steroid alcohols. The reaction allows the conversion of alcohols with alkyl or aryl carboxylic acids in the presence of diethyl azodicarboxylate and triphenylphosphine (Ph_3_P). The result is an alkyl- or aryl carboxylic ester of the alcohol with inverted configuration (Mitsunobu, 1981). Here, we describe the Mitsunobu reaction for 19-nortestosterone (**1**) utilizing 2,4-, or 3,5-dinitrobenzoic acid in the presence of diethyl azodicarboxylate and Ph_3_P in toluene at 80°C leading to the corresponding 17α-19-nortestosterone-17-yl 2′,4′- or 3′,5′-dinitrobenzoate (**6** or **7**, respectively; **Figure 1**). Reacting compound **1** with isopropyl halides under the same conditions produces the corresponding 17α-chloro-, bromo-, and iodo-19-nortestosterone (**3**−**5**, respectively). Hydrolyzing compounds **6** or **7** in methanol, in the presence of NaOCH_3_ yields 17α-19-nortestosterone (**2**).

### Antiproliferative Properties of 19-Nortestosterone Derivatives

The antiproliferative activities of the test compounds were determined by MTT assay on a panel of adherent gynecological cancer cell lines (**Table 1**). Nandrolone (**1**) as reported previously, exerted a considerable antiproliferative effect against HeLa cells. Compounds **2**, **6**, and **7** exerted no remarkable antiproliferative action against the gynecological cancer cell lines. 17α-Halogen derivatives (**3−5**) exerted a pronounced antiproliferative effect against HeLa cells, while they did not elicit any notable influence on the remaining cell lines including fibroblasts. These derivatives had lower IC_50_ values on HeLa cells than that of the reference agent CIS. Compound **3** proved to be the most potent antiproliferative agent, characterized by an effect size comparable to that of **1**. Cancer selectivity of the potent compounds was determined by the same method using human fibroblasts (MRC-5) and non-cancerous immortalized, mammary gland derived epithelial (hTERT-HME1) cells. None of the test agents exhibited a considerable growth inhibitory effect against intact fibroblasts up to a concentration of 30 μM. Compound **1** had no pronounced action on immortalized epithelial cells, while compound **3** inhibited the growth of these cells with an IC_50_ value approximately four times higher than that obtained on HeLa cells. Compound **3** exerted the most explicit tumor selectivity, showing a substantially weaker effect on non-cancerous cells than CIS. Due to their potent and selective antiproliferative actions, compound **3** and nandrolone (**1**) were selected for further investigations to characterize their mechanism of action and assess their hormonal effect.

**Table 1:** Antiproliferative effects of the synthesized compounds (1−7) on human cell lines.

Comp.	Conc. (μM)	Growth inhibition (%) ± SEM [calculated IC_50_ value (μM)]^a^									
		HeLa	SiHa	C33A	A2780	MCF-7	MDA-MB-231	MDA-MB-361	T47D	MRC-5	hTERT-HME1
**1**^b^	10	99.5 ± 0.2	−^c^	−	−	−	−	−	−	5.4 ± 1.2	16.9 ± 1.2
	30	99.5 ± 0.3	20.7 ± 2.6	25.2 ± 1.7	−	−	21.4 ± 1.2	−	−	11.3 ± 0.5	37.1 + 1.0
		[0.65]									
**2**^b^	10	28.1 ± 4.0	−	−	−	−	−	−	−	n.d.^d^	n.d.
	30	37.9 ± 3.4	−	−	−	27.7 ± 3.7	−	−	−		
**3**	10	95.9 ± 0.3	−	−	−	−	−	−	−	4.3 ± 3.7	76.2 ± 0.5
	30	95.1 ± 0.5	26.0 ± 2.3	61.7 ± 1.7	32.7 ± 1.0	36.9 ± 2.0	−	21.8 ± 2.5	−	8.0 ± 2.3	99.9 ± 0.1
		[1.21]									[4.63]
**4**	10	94.4 ± 0.7	−	−	−	−	−	−	−	11.9 ± 2.3	n.d.
	30	94.1 ± 0.5	−	−	54.4 ± 1.6	−	−	−	−	20.4 ± 2.1	
		[1.69]									
**5**	10	95.2 ± 0.4	−	−	27.7 ± 1.3	−	−	−	−	13.1 ± 1.9	n.d.
	30	95.8 ± 0.2	−	−	61.6 ± 1.8	−	−	−	−	13.8 ± 2.0	
		[1.49]									
**6**	10	32.0 ± 2.9	−	−	−	−	−	−	−	n.d.	n.d.
	30	26.5 ± 2.3	−	−	31.9 ± 2.6	35.9 ± 1.4	−	−	47.2 ± 2.6		
**7**	10	26.6 ± 1.8	−	−	49.2 ± 0.9	25.9 ± 3.1	−	−	−	n.d.	n.d.
	30	21.5 ± 1.9	−	−	58.1 ± 2.0	37.7 ± 3.4	−	22.0 ± 1.6	−		
CIS^e^	10	42.6 ± 2.3	88.6 ± 0.5	83.8 ± 0.8	83.6 ± 1.2	66.9 ± 1.8	−	67.5 ± 1.0	51.0 ± 2.0	60.3 ± 3.3	97.7 ± 0.3
	30	99.9 ± 0.3	90.2 ± 1.8	93.9 ± 0.6	95.0 ± 0.3	96.8 ± 0.4	71.5 ± 1.2	87.8 ± 1.1	55.0 ± 1.5	61.9 ± 1.0	99.1 ± 0.3
		12.43	7.84	1.77	1.30	5.78	19.13	3.74	9.78	6.19	[2.45]

*^a^IC_50_ values were calculated when the growth inhibition value for a compound exceeded 75% at a concentration of 30 μM. See Supplementary Figure S1 for representative concentration-response curves. ^b^Data previously reported (Schneider et al., 2016). ^c^Inhibition values less than 20% are not presented. ^d^n.d., not determined. ^e^Reference agent cisplatin.*

### Cytotoxic Activity

**FIGURE 2:**
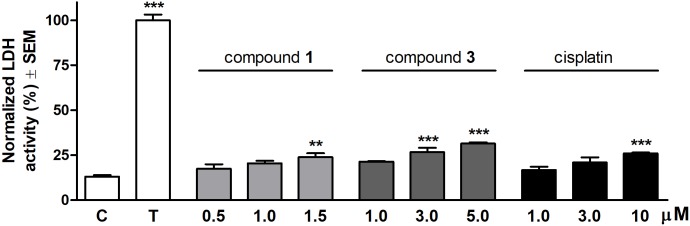
Cytotoxic effects of compounds 1, 3 and cisplatin on HeLa cells after 24 h treatment. The effect of Triton X-100 (T) was considered 100%. Results are mean values ± SEM of the data from two separate measurements, *n* = 4. ^∗^*p* < 0.05, ^∗∗^*p* < 0.01, and ^∗∗∗^*p* < 0.001 compared to untreated control (C).

Cytotoxic properties of the selected compounds were ascertained by measuring LDH activity resulting from cell membrane damage. Each molecule exerted a concentration-dependent increase of LDH activity compared to the untreated control after 24 h treatment (**Figure 2**). Compound **1** elicited a substantial LDH release at a concentration of 1.5 μM. The effect of compound **3** proved to be significant when applied at concentrations above its IC_50_ values (3.0 or 5.0 μM). None of the test agents induced an LDH activity comparable to the maximum LDH release triggered by detergent Triton X-100.

### Cell Cycle Analysis

**FIGURE 3:**
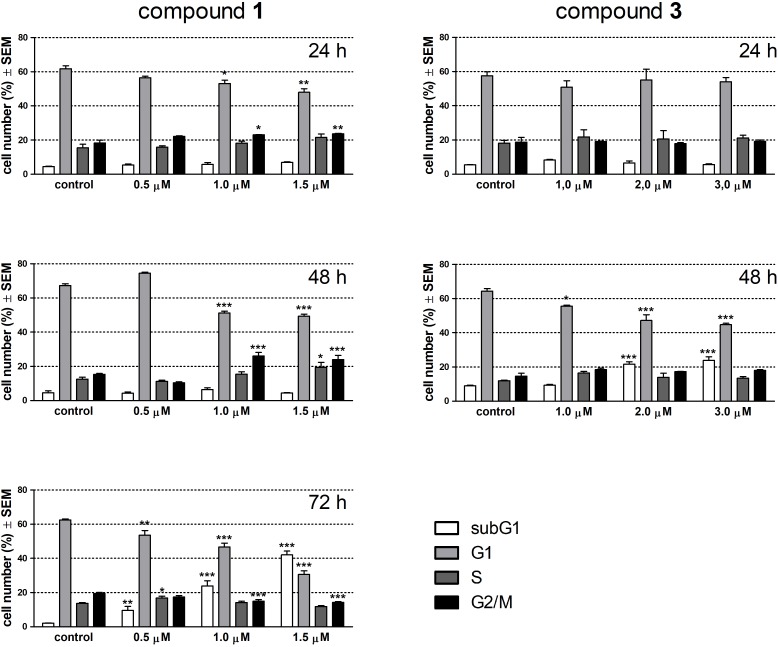
Effects of compounds 1 and 3 on cell cycle phase distribution of HeLa cells determined by flow cytometry after incubation for 24, 48, or 72 h. Results are mean values ± SEM of the data from two independent measurements, *n* = 6. ^∗^*p* < 0.05, ^∗∗^*p* < 0.01, and ^∗∗∗^*p* < 0.001 compared to untreated control.

Alterations in cell cycle and apoptotic fragmentations were determined by flow cytometry after treatment with the test compounds for 24 and 48 h. Since **1** elicited no change in the subG1 population at these time points this agent was re-tested after 72 h incubation. Treatment with **1** for 24 h resulted in a concentration-dependent and significant decrease in the G1 and a moderate but significant increase in the G2/M phase cell population (**Figure 3**). After 48 h of exposure these changes became more pronounced, and completed with the elevated ratio of the S phase cell population in the presence of 1.5 μM of **1**. A longer incubation time (72 h) with **1** elicited a substantial disturbance in the cell phase distribution and a concentration-dependent accumulation of hypodiploid cells indicating apoptotic nuclear fragmentation. Compound **3** did not have any remarkable effect on cell cycle after 24 h exposure, while a longer incubation time (48 h) resulted in a substantially elevated increase of the subG1 population at the expense of G1 cells.

### Morphological Changes

**FIGURE 4:**
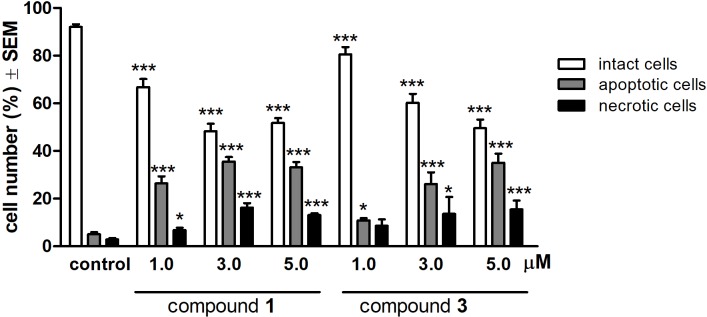
Qualitative evaluation of fluorescent double staining of HeLa cells after 24 h treatment with compounds 1 or 3. ^∗^*p* < 0.05 and ^∗∗∗^*p* < 0.001 compared to untreated control (C). See Supplementary Figure S2 for representative pictures.

To characterize the morphological features of the apoptosis induced by compounds **1** and **3** HeLa cells were examined by fluorescent microscopy after 24 h treatment with three different concentrations (1.0, 3.0, or 5.0 μM) of the test compounds. For the quantitative analysis, cells with intact, apoptotic and necrotic morphological features were labeled, and the ratios of different morphologies were calculated. Treatments with **1** and **3** resulted in a substantial and concentration-dependent increase in both the apoptotic and necrotic cell populations, at the expense of the intact population (**Figure 4**).

### Induction of Apoptotic Enzymes

**FIGURE 5:**
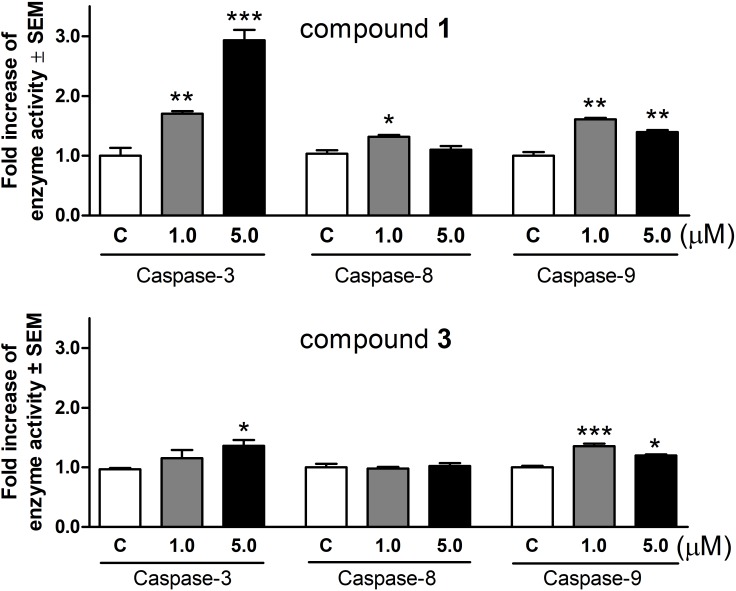
Activation of caspase-3, caspase-8, and caspase-9 enzymes in HeLa cells after incubation with compounds 1 and 3 for 72 or 24 h, respectively. Results are mean values ± SEM of the data from two independent measurements, *n* = 6. ^∗^*p* < 0.05, ^∗∗^*p* < 0.01, and ^∗∗∗^*p* < 0.001 compared to untreated control.

Based on the above results, changes of the activities of caspase-3, caspase-8, and caspase-9 were determined using a colorimetric assay. After treatment with **1** for 72 h, the activity of executive caspase-3 increased significantly and in a concentration-dependent manner (**Figure 5**). Under the same experimental conditions **1** also activated the initiator caspases, although the induction of caspase-8 was less pronounced. Compound **3** enhanced caspase-3 activity at 5.0 μM even after a shorter incubation period (24 h). Caspase-9 activity was also significantly elevated, while there was no change in the function of caspase-8.

### Tubulin Polymerization

**FIGURE 6:**
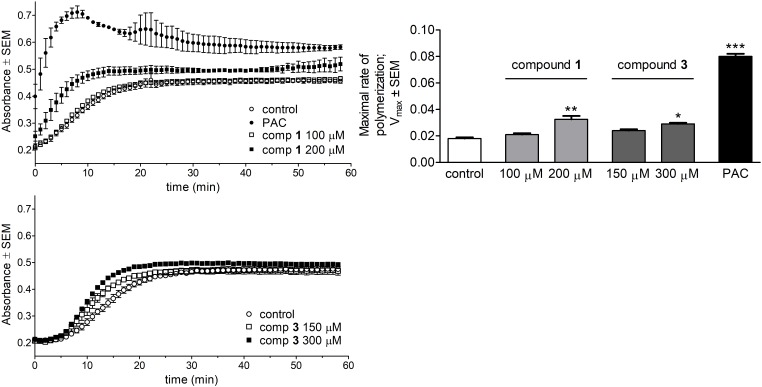
Direct effects of compounds 1 and 3 on tubulin polymerization. Kinetic curves characterizing tubulin polymerization in the presence of 1, 3 or paclitaxel (PAC) were recorded after 58 min kinetic measurements (Left). Direct effects of the test agents on the maximal rate of polymerization were evaluated (Right). Results are mean values ± SEM of the data from two independent measurements, *n* = 4. ^∗^*p* < 0.05, ^∗∗^*p* < 0.01, and ^∗∗∗^*p* < 0.001 compared to untreated control sample.

The direct effect of the test compounds on microtubule formation was determined by a specific photometric assay in a cell-free system. The tested concentrations of the compounds were chosen based on their calculated IC_50_ values according to the recommendation of the manufacturer. Both compounds **1** and **3** induced a significant acceleration of tubulin polymerization compared to untreated control samples (**Figure 6**). Calculated values of maximal rate of tubulin polymerization (*V*_max_) were elevated compared to control, although none of these *V*_max_ values were comparable to that of the reference agent PAC.

### Hormonal Effect

**FIGURE 7:**
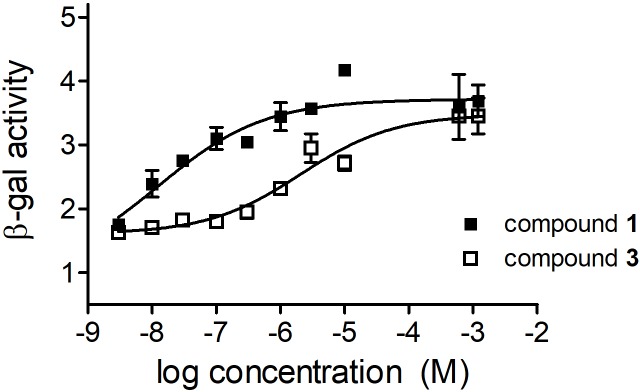
Effects of compounds 1 and 3 in the yeast-based androgenic reporter assay. Results are mean values ± SEM of the data from two independent measurements, *n* = 4.

Since residual hormonal activity of a potential sterane lead compound is a crucial aspect of further drug development, the androgenic properties of the most promising agents were investigated by a yeast-based reporter assay. As **1** is a well-characterized androgen, it was used as a reference agent (Bergink et al., 1985). According to our results **3** exerts a substantially lower hormonal activity, with no relevant action unless applied in extremely high concentrations (**Figure 7**). Calculated EC_50_ values of **1** and **3** differed by approximately 2 orders of magnitude (3.43 × 10^-8^ and 3.80 × 10^-6^ M, respectively). Compound **3** exhibited no antagonistic activity in the assay system (data not shown).

## Discussion

Various biological effects of compounds with an androstane skeleton mostly stem from their endocrine disruptor properties, thus the medical use of androgens is mainly limited to androgen replacement and androgen deprivation therapies, including the management of some hormone dependent malignancies (Wadosky and Koochekpour, 2016). Beyond their approved medical applications, numerous androgenic anabolic steroids are utilized illegally to enhance physical performance, a risky use often accompanied by serious adverse effects. Recently, numerous androstanes with anticancer potential have been described, and the importance of androstane compounds and their derivatives in the research and development of steroid-based anticancer agents for hormone-independent malignancies is continuously increasing (Frank and Schneider, 2013; Wadosky and Koochekpour, 2016). Although several 19-nortestosterone derivatives have been identified as potent anticancer agents (e.g., mibolerone, tibolone, gestodene), most of them have pronounced hormonal properties involved in their therapeutic action, as well as in their undesired adverse effects (Saito et al., 2016). In a previous research, we investigated a set of newly synthesized 19-nortestosterone analogs, and reported that some of them exhibited a moderate antiproliferative activity. That study of our research group revealed that the widely known 19-nortestosterone analog **1** (nandrolone) has a potent and selective antiproliferative effect against cervical carcinoma cells positive for type 18 of human papilloma virus (HPV-18) (Schneider et al., 2016).

The aim of our present study was to synthesize and investigate a set of novel 19-nortestosterone derivatives with improved antiproliferative properties and limited hormonal activities. Three of the novel compounds (**3**−**5**) were found to exhibit a pronounced antiproliferative effect against HeLa cells (calculated IC_50_ values: 1.21−1.69 μM), while exerting a negligible or lower impact on other cell lines including intact fibroblasts (MRC-5) and the immortalized mammary gland epithelial cell line (hTERT-HME1). In contrast, dinitrobenzoates (**6** and **7**) appeared to be ineffective in terms of growth inhibition of cancer cells. The most potent compound, **3** was further investigated to describe its possible mechanism of action. A well-known androgen, **1** a with similar antiproliferative capacity was utilized as a steroidal reference agent.

The antiproliferative property of a compound is typically reflected by a disturbance induced in cell cycle distribution. These changes in cell cycle phases inform about the probable mechanism of the antiproliferative action. Both **1** and **3** caused a cell cycle disturbance characterized by the accumulation of hypodiploid (subG1) cells at the expense of the G1 population.

The increase of hypodiploid cell populations can be regarded as an evidence for proapoptotic properties of the test compounds. Alterations in cell cycle during physiological conditions usually lead to induction of programmed cell death. Activation of the apoptotic machinery, selectively in cancer cells without a substantial necrotizing effect is one of the most desirable characteristics of a promising anticancer agent (Tolomeo and Simoni, 2002). Some steroidal compounds with anticancer activity (e.g., 2-methoxyestradiol, D-homoestrone, a D-secoestrone-triazole analog) have been described as efficacious inducers of programmed cell death in cancer cells (Li et al., 2004; Minorics et al., 2015; Bózsity et al., 2017). Therefore, the demonstration of apoptosis induction was a basic feature of our study. We utilized fluorescent microscopy and observed the characteristic features of apoptosis elicited by the test agents in a concentration-dependent manner.

Caspase enzymes are crucial implementers of the apoptotic program executed by downstream effector caspases such as caspase-3. Caspase-9 is the major enzyme involved in the initiation of the intrinsic apoptotic pathway, while caspase-8 plays an essential role in the extrinsic pathway of the apoptotic process (Hajra and Liu, 2004). Treatments with the test compounds resulted in a significant elevation in the activity of caspase-3, reflecting the activation of apoptotic cell death. Both agents elicited a considerable increase in activity of caspase-9 at both concentrations tested (1 and 5 μM) without expected concentration-dependency. The exact reason for this is not elucidated, but based on the results of our fluorescent microscopy analysis, necrotic cell death induced by the higher concentration could be a plausible explanation. Based on these findings activation of the intrinsic apoptotic pathway is hypothesized. Although, the activity of caspase-8 was slightly but significantly increased by **1** at a concentration of 1 μM, this limited change seems to be inefficient to indicate a dominant role of the extrinsic apoptotic pathway.

Previous studies revealed that some proapoptotic steroidal compounds induced a pronounced cell cycle arrest via a direct influence on tubulin polymerization during mitosis. The inhibitory effect of 2-methoxyestradiol on microtubule formation resulting from its interaction with the colchicine-binding site of β-tubulin has also been reported (Peyrat et al., 2012). Thus, a possible direct influence of **1** and **3** on the polymerization of tubulin heterodimers in a cell-free system was also investigated.

Both of our test agents elicited a concentration-dependent acceleration of the polymerization reaction as reflected by significantly increased *V*_max_ values. This molecular behavior indicates a possible PAC-like microtubule stabilizing effect of the test compounds which may contribute to cell cycle arrest and lead to the induction of the apoptotic machinery.

The possible androgenic activity of a novel steroid-based agent may implicate a source of potential adverse reactions limiting its therapeutic value. Receptor binding properties of **1** and 5α-dihydrotestosterone are indistinguishable by a radio ligand assay using androgen receptors prepared from rat prostate and MCF-t cells (Bergink et al., 1985). Therefore, **1** can be utilized as a reference agent when novel compounds with possible androgenic properties are characterized. Compound **3** was detected to possess a substantially lower androgenic activity compared to **1**.

Moreover, **3** had no androgen antagonistic properties when tested in the presence 5α-dihydrotestosterone. This hormonal neutrality of **3** could be explained by the α-position of chlorine on the ring D of the skeleton. The substituent at position 17 determines receptor binding, and only the β configuration is favored. Consequently, 17α-testosterone has virtually no affinity for the receptor (Fragkaki et al., 2009).

Although mediated by the same nuclear receptor, androgenic and anabolic actions of a ligand can be partially dissociated depending on the tissue expression of crucial metabolic enzymes including 5α-reductase. While the androgenic action may be disadvantageous and may limit the development of a drug candidate, an anabolic or “myotropic” property could theoretically be advantageous when a chronic or devastating disease is treated (Tóth and Zakár, 1982).

Beside the well-characterized action of androgens mediated via androgen response elements within the DNA, the presented 19-nortestosterone analogs may also interact with membrane-associated androgen receptors (Kampa et al., 2006). This latter action could be of special importance since the stimulation of these receptors elicits an increase in the intracellular free zinc concentration accompanied by induction of apoptosis in cancer cell (Thomas et al., 2014).Two further membrane-bound proteins have been recently reported as potential non-genomic receptors for androgens and related steroids. One of them is the putative G-protein coupled receptor GPRC6A, an amino acid, calcium, and osteocalcin sensing receptor. The interaction of this receptor with testosterone is reported to result in increased phosphorylation of extracellular signal-regulated kinases in human embryonic kidney cells expressing the GPRC6A protein (Pi et al., 2010).

Oxoeicosanoid receptor 1 (OXER1) is a membrane receptor for the arachidonic acid metabolite 5-oxoeicosatetraenoic acid (5-oxoETE) and serves as a binding site for testosterone in prostate cancer cells. The steroid testosterone is reported to antagonize the action of the natural agonist 5-oxoETE on the intracellular cAMP production, and the interaction between testosterone and OXER1 was confirmed by an *in silico* molecular docking study as well (Kalyvianaki et al., 2017).

Since apoptosis and cell growth are indirectly involved in the signal mechanism of these non-genomic receptors, an action mediated by membrane-bound steroid receptors may contribute to the overall effects of the presented compounds.

In summary, our present results demonstrated that three of a set of newly synthesized 19-nortestosterone exhibit a pronounced antiproliferative activity against cervical carcinoma cells with lower influence on fibroblasts and a modest action on non-cancerous immortalized epithelial cells. The most potent agent **3** is characterized by a moderate cytotoxic effect, elicits cell cycle disturbance and induces the mitochondrial pathway of apoptosis. As a possible molecular mechanism of these actions, a PAC-like microtubule-stabilizing property is suggested based on its direct effect on the microtubular system.

Based on our present findings, the 19-nortestosterone backbone with a 17α-halogen substitution provides an excellent skeleton for designing novel antiproliferative steroidal compounds with negligible androgenic activity.

## Author Contributions

AG, AK, and EK performed the experiments. IO and AS analyzed the data. RM, EM, GS, and IZ were involved in experiment planning and supervision. AG, GS, and IZ wrote the manuscript.

## Conflict of Interest Statement

The authors declare that the research was conducted in the absence of any commercial or financial relationships that could be construed as a potential conflict of interest.

## References

[B1] Acharya P. C. Bansal R. (2014). Synthesis and antiproliferative activity of some androstene oximes and their O-Alkylated derivatives. *Arch. Pharm.* 347 193–199. 10.1002/ardp.201300216 24343881

[B2] Ajdukovic J. J. Djurendic E. A. Petri E. T. Klisuric O. R. Celic A. S. Sakac M. N. (2013). 17(E)-picolinylidene androstane derivatives as potential inhibitors of prostate cancer cell growth: antiproliferative activity and molecular docking studies. *Bioorg. Med. Chem.* 21 7257–7266. 10.1016/j.bmc.2013.09.063 24148837

[B3] Ajdukovic J. J. Penov Gasi K. M. Jakimov D. S. Klisuric O. R. Jovanovic-Santa S. S. Sakac M. N. (2015). Synthesis, structural analysis and antitumor activity of novel 17alpha-picolyl and 17(E)-picolinylidene A-modified androstane derivatives. *Bioorg. Med. Chem.* 23 1557–1568. 10.1016/j.bmc.2015.02.001 25737400

[B4] Banday A. H. Mir B. P. Lone I. H. Suri K. A. Kumar H. M. (2010). Studies on novel D-ring substituted steroidal pyrazolines as potential anticancer agents. *Steroids* 75 805–809. 10.1016/j.steroids.2010.02.014 20206644

[B5] Bergink E. W. Janssen P. S. Turpijn E. W. van der Vies J. (1985). Comparison of the receptor binding properties of nandrolone and testosterone under in vitro and in vivo conditions. *J. Steroid Biochem.* 22 831–836. 10.1016/0022-4731(85)90293-6 4021486

[B6] Bózsity N. Minorics R. Szabó J. Mernyák E. Schneider G. Wölfling J. (2017). Mechanism of antiproliferative action of a new d-secoestrone-triazole derivative in cervical cancer cells and its effect on cancer cell motility. *J. Steroid Biochem. Mol. Biol.* 165 247–257. 10.1016/j.jsbmb.2016.06.013 27363663

[B7] Campagnoli C. Clavel-Chapelon F. Kaaks R. Peris C. Berrino F. (2005). Progestins and progesterone in hormone replacement therapy and the risk of breast cancer. *J. Steroid Biochem. Mol. Biol.* 96 95–108. 10.1016/j.jsbmb.2005.02.014 15908197 PMC1974841

[B8] Cops E. J. Bianco-Miotto T. Moore N. L. Clarke C. L. Birrell S. N. Butler L. M. (2008). Antiproliferative actions of the synthetic androgen, mibolerone, in breast cancer cells are mediated by both androgen and progesterone receptors. *J. Steroid Biochem. Mol. Biol.* 110 236–243. 10.1016/j.jsbmb.2007.10.014 18515094

[B9] Cui J. G. Liu L. Zhao D. D. Gan C. F. Huang X. Xiao Q. (2015). Synthesis, characterization and antitumor activities of some steroidal derivatives with side chain of 17-hydrazone aromatic heterocycle. *Steroids* 95 32–38. 10.1016/j.steroids.2015.01.002 25578734

[B10] Erel C. T. Senturk L. M. Kaleli S. (2006). Tibolone and breast cancer. *Postgrad. Med. J.* 82 658–662. 10.1136/pgmj.2005.037184 17068276 PMC2653908

[B11] Fitzmaurice C. Allen C. Barber R. M. Barregard L. Bhutta Z. A. Brenner H. (2017). Global, regional, and national cancer incidence, mortality, years of life lost, years lived with disability, and disability-adjusted life-years for 32 cancer groups, 1990 to 2015: a systematic analysis for the global burden of disease study. *JAMA Oncol.* 3 524–548. 10.1001/jamaoncol.2016.5688 27918777 PMC6103527

[B12] Fotsis T. Zhang Y. Pepper M. S. Adlercreutz H. Montesano R. Nawroth P. P. (1994). The endogenous oestrogen metabolite 2-methoxyoestradiol inhibits angiogenesis and suppresses tumour growth. *Nature* 368 237–239. 10.1038/368237a0 7511798

[B13] Fragkaki A. G. Angelis Y. S. Koupparis M. Tsantili-Kakoulidou A. Kokotos G. Georgakopoulos C. (2009). Structural characteristics of anabolic androgenic steroids contributing to binding to the androgen receptor and to their anabolic and androgenic activities. Applied modifications in the steroidal structure. *Steroids* 74 172–197. 10.1016/j.steroids.2008.10.016 19028512

[B14] Frank E. Schneider G. (2013). Synthesis of sex hormone-derived modified steroids possessing antiproliferative activity. *J. Steroid Biochem. Mol. Biol.* 137 301–315. 10.1016/j.jsbmb.2013.02.018 23499871

[B15] Franke H. R. Vermes I. (2003). Differential effects of progestogens on breast cancer cell lines. *Maturitas* 46(Suppl. 1), S55–S58. 10.1016/j.maturitas.2003.09.019 14670646

[B16] Hajra K. M. Liu J. R. (2004). Apoptosome dysfunction in human cancer. *Apoptosis* 9 691–704. 10.1023/B:APPT.0000045786.98031.1d 15505412

[B17] Iványi Z. Szabó N. Huber J. Wölfling J. Zupkó I. Szécsi M. (2012). Synthesis of D-ring-substituted (5’R)- and (5’S)-17b-pyrazolinylandrostene epimers and comparison of their potential anticancer activities. *Steroids* 77 566–574. 10.1016/j.steroids.2012.02.001 22342542

[B18] Jakimov D. S. Kojic V. V. Aleksic L. D. Bogdanovic G. M. Ajdukovic J. J. Djurendic E. A. (2015). Androstane derivatives induce apoptotic death in MDA-MB-231 breast cancer cells. *Bioorg. Med. Chem.* 23 7189–7198. 10.1016/j.bmc.2015.10.015 26494582

[B19] Jordan M. A. Wendell K. Gardiner S. Derry W. B. Copp H. Wilson L. (1996). Mitotic block induced in HeLa cells by low concentrations of paclitaxel (Taxol) results in abnormal mitotic exit and apoptotic cell death. *Cancer Res.* 56 816–825. 8631019

[B20] Kalyvianaki K. Gebhart V. Peroulis N. Panagiotopoulou C. Kiagiadaki F. Pediaditakis I. (2017). Antagonizing effects of membrane-acting androgens on the eicosanoid receptor OXER1 in prostate cancer. *Sci. Rep.* 7:44418. 10.1038/srep44418 28290516 PMC5349529

[B21] Kampa M. Kogia C. Theodoropoulos P. A. Anezinis P. Charalampopoulos I. Papakonstanti E. A. (2006). Activation of membrane androgen receptors potentiates the antiproliferative effects of paclitaxel on human prostate cancer cells. *Mol. Cancer Ther.* 5 1342–1351. 10.1158/1535-7163.MCT-05-0527 16731768

[B22] Kloosterboer H. J. Schoonen W. G. Deckers G. H. Klijn J. G. (1994). Effects of progestagens and Org OD14 in in vitro and in vivo tumor models. *J. Steroid Biochem. Mol. Biol.* 49 311–318. 10.1016/0960-0760(94)90273-9 8043494

[B23] Koduru S. Grierson D. S. van de Venter M. Afolayan A. J. (2007). Anticancer activity of steroid alkaloids isolated from *Solanum aculeastrum*. *Pharm. Biol.* 45 613–618. 10.1080/13880200701538690

[B24] LaVallee T. M. Zhan X. H. Herbstritt C. J. Kough E. C. Green S. J. Pribluda V. S. (2002). 2-Methoxyestradiol inhibits proliferation and induces apoptosis independently of estrogen receptors alpha and beta. *Cancer Res.* 62 3691–3697. 12097276

[B25] Li L. Bu S. Backstrom T. Landstrom M. Ulmsten U. Fu X. (2004). Induction of apoptosis and G2/M arrest by 2-methoxyestradiol in human cervical cancer HeLaS3 cells. *Anticancer Res.* 24 873–880. 15161040

[B26] Lin S. X. Chen J. Mazumdar M. Poirier D. Wang C. Azzi A. (2010). Molecular therapy of breast cancer: progress and future directions. *Nat. Rev. Endocrinol.* 6 485–493. 10.1038/nrendo.2010.92 20644568

[B27] Mijatovic T. Van Quaquebeke E. Delest B. Debeir O. Darro F. Kiss R. (2007). Cardiotonic steroids on the road to anti-cancer therapy. *Biochim. Biophys. Acta* 1776 32–57. 10.1016/j.bbcan.2007.06.002 17706876

[B28] Minami T. Kosugi K. Suganuma I. Yamanaka K. Kusuki I. Oyama T. (2013). Antiproliferative and apoptotic effects of norethisterone on endometriotic stromal cells in vitro. *Eur. J. Obstet. Gynecol. Reprod. Biol.* 166 76–80. 10.1016/j.ejogrb.2012.08.023 22964137

[B29] Minorics R. Bózsity N. Molnár J. Wölfling J. Mernyák E. Schneider G. (2015). A molecular understanding of d-homoestrone-induced G2/M cell cycle arrest in HeLa human cervical carcinoma cells. *J. Cell Mol. Med.* 19 2365–2374. 10.1111/jcmm.12587 26228523 PMC4594678

[B30] Mitsunobu O. (1981). The use of diethyl azodicarboxylate and triphenylphosphine in synthesis and transformation of natural products. *Synthesis* 1981 1–28. 10.1055/s-1981-29317

[B31] Miyashita M. Koga K. Takamura M. Izumi G. Nagai M. Harada M. (2014). Dienogest reduces proliferation, aromatase expression and angiogenesis, and increases apoptosis in human endometriosis. *Gynecol. Endocrinol.* 30 644–648. 10.3109/09513590.2014.911279 24805834

[B32] Mohamed Z. H. El-Koussi N. A. Mahfouz N. M. Youssef A. F. Abdel Jaleel G. A. Shouman S. A. (2015). Cu (I) catalyzed alkyne-azide 1,3-dipolar cycloaddition (CuAAC): synthesis of 17alpha-[1-(substituted phenyl)-1,2,3-triazol-4-yl]-19-nor-testosterone-17beta-yl acetates targeting progestational and antipro-liferative activities. *Eur. J. Med. Chem.* 97 75–82. 10.1016/j.ejmech.2015.04.045 25942354

[B33] Mosmann T. (1983). Rapid colorimetric assay for cellular growth and survival: application to proliferation and cytotoxicity assays. *J. Immunol. Methods* 65 55–63. 10.1016/0022-1759(83)90303-4 6606682

[B34] Newman R. A. Yang P. Pawlus A. D. Block K. I. (2008). Cardiac glycosides as novel cancer therapeutic agents. *Mol. Interv.* 8 36–49. 10.1124/mi.8.1.8 18332483

[B35] Peyrat J. F. Brion J. D. Alami M. (2012). Synthetic 2-methoxyestradiol derivatives: structure-activity relationships. *Curr. Med. Chem.* 19 4142–4156. 10.2174/092986712802430072 22709003

[B36] Pi M. Parrill A. L. Quarles L. D. (2010). GPRC6A mediates the non-genomic effects of steroids. *J. Biol. Chem.* 285 39953–39964. 10.1074/jbc.M110.158063 20947496 PMC3000977

[B37] Punjabi S. Cook L. J. Kersey P. Marks R. Cerio R. (2008). Solasodine glycoalkaloids: a novel topical therapy for basal cell carcinoma. A double-blind, randomized, placebo-controlled, parallel group, multicenter study. *Int. J. Dermatol.* 47 78–82. 10.1111/j.1365-4632.2007.03363.x 18173610

[B38] Royer P. A. Jones K. P. (2014). Progestins for contraception: modern delivery systems and novel formulations. *Clin. Obstet. Gynecol.* 57 644–658. 10.1097/GRF.0000000000000072 25314087

[B39] Saito F. Tashiro H. Yamaguchi M. Honda R. Ohba T. Suzuki A. (2016). Development of a mouse model for testing therapeutic agents: the anticancer effect of dienogest on endometrial neoplasms. *Gynecol. Endocrinol.* 32 403–407. 10.3109/09513590.2015.1124411 26680656

[B40] Schneider G. Kiss A. Mernyák E. Benke Z. Wölfling J. Frank É. (2016). Stereocontrolled synthesis of the four 16-hydroxymethyl-19-nortestosterone isomers and their antiproliferative activities. *Steroids* 105 113–120. 10.1016/j.steroids.2015.12.003 26686898

[B41] Sharifi N. Gulley J. L. Dahut W. L. (2010). An update on androgen deprivation therapy for prostate cancer. *Endocr. Relat. Cancer* 17 R305–R315. 10.1677/ERC-10-0187 20861285 PMC3461824

[B42] Thomas P. Pang Y. Dong J. Berg A. H. (2014). Identification and characterization of membrane androgen receptors in the ZIP9 zinc transporter subfamily: II. Role of human ZIP9 in testosterone-induced prostate and breast cancer cell apoptosis. *Endocrinology* 155 4250–4265. 10.1210/en.2014-1201 25014355 PMC4197988

[B43] Tolomeo M. Simoni D. (2002). Drug resistance and apoptosis in cancer treatment: development of new apoptosis-inducing agents active in drug resistant malignancies. *Curr. Med. Chem. Anticancer Agents* 2 387–401. 10.2174/1568011024606361 12678739

[B44] Tóth M. Zakár T. (1982). Relative binding affinities of testosterone, 19-nortestosterone and their 5 alpha-reduced derivatives to the androgen receptor and to other androgen-binding proteins: a suggested role of 5 alpha-reductive steroid metabolism in the dissociation of ”myotropic” and ”androgenic” activities of 19-nortestosterone. *J. Steroid Biochem.* 17 653–660. 10.1016/0022-4731(82)90567-2 6891012

[B45] Wadosky K. M. Koochekpour S. (2016). Molecular mechanisms underlying resistance to androgen deprivation therapy in prostate cancer. *Oncotarget* 7 64447–64470. 10.18632/oncotarget.10901 27487144 PMC5325456

[B46] Yue T. L. Wang X. Louden C. S. Gupta S. Pillarisetti K. Gu J. L. (1997). 2-Methoxyestradiol, an endogenous estrogen metabolite, induces apoptosis in endothelial cells and inhibits angiogenesis: possible role for stress-activated protein kinase signaling pathway and Fas expression. *Mol. Pharmacol.* 51 951–962. 10.1124/mol.51.6.951 9187261

