# Supplementary material for: Antiproliferative Properties of Newly Synthesized 19-Nortestosterone Analogs Without Substantial Androgenic Activity
Source: Front Pharmacol. 2018 Jul 27;9:825. doi: 10.3389/fphar.2018.00825 (PMC6072853; doi:10.3389/fphar.2018.00825)
Supplement: Supplementary file 1 [file Data_Sheet_1.pdf]

Supplementary Material

Antiproliferative properties of newly synthesized 19-nortestosterone analogs without substantial androgenic activity

András Gyovai[1], Renáta Minorics[1], Anita Kiss[2], Erzsébet Mernyák[2], Gyula Schneider[2], András Szekeres[3], Erika Kerekes[3], Imre Ocsovszki[4], István Zupkó[1,5]*

[1]Department of Pharmacodynamics and Biopharmacy, University of Szeged, Szeged, Hungary

[2]Department of Organic Chemistry, University of Szeged, Szeged, Hungary

[3]Department of Microbiology, University of Szeged, Szeged, Hungary

[4]Department of Biochemistry, University of Szeged, Szeged, Hungary

[5]Interdisciplinary Centre for Natural Products, University of Szeged, Szeged, Hungary

Correspondence: István Zupkó PhD zupko@pharm.u-szeged.hu

Experimental

Melting points were determined on a Kofler block. Optical rotation were measured in chloroform (c = 1) on a Polamat-A (Zeiss, Jena) polarimeter and are given in units 10^{-1} deg cm^2 g^{-1}. NMR spectra were recorded on a Bruker AM 500 instrument. Chemical shift (δ) are given in ppm, and coupling constants (J) in Hz. Elemental analysis data were determined with a Perkin-Elmer CHN analyser model 2400. For all new compounds satisfactory elemental analysis were obtained.

1. General procedure

19-nortestosterone (1 mmol, 274.46 mg), 656 mg of triphenylphosphane (2.5 mmol), and 2.5 mmol alkyl halides or substituted benzoic acids were suspended in 15 ml dry toluene in a 50 ml, three-necked, round-bottomed flask equipped with thermometer, dropping funnel, and a reflux condenser. To the suspension 436 mg (2.5 mmol) of diethyl azodicarboxylate was then

added dropwise at room temperature. The suspension cleared to give a yellow-orange solution and became slightly warm. The reaction mixture was kept at 80 °C for 1.5 h, the solvent was then removed under *vacuum*, and the residue was subjected to chromatography on silicagel column.

1.1. 17α-19-nortestosterone-17-yl 2',4'-dinitrobenzoate (6)

Compound **1** (1 mmol) and 530 mg (2.5 mmol) 2,4-dinitrobenzoic acid was treated in 15 ml toluene as described in the general procedure. The resulting crude product was chromatographed on a silica gel column with CH_2Cl_2, yielding pure **6** (280 mg, 59%). Mp: 136–138 °C, $[\alpha]_D^{20}$ – 39 (*c* = 1 in $CHCl_3$). (Found: C, 63.95; H, 6.25. $C_{25}H_{28}N_2O_7$ (468.50) requires: C, 64.09; H, 6.02 %). ^{1}H-NMR (500 MHz, $CDCl_3$) δ [ppm] = 0.87 (s, 3H, 18-H_3), 5.08 (d, 1H, *J* = 6.0 Hz, 17-H), 5.82 (s, 1H, 4-H), 7.95 (d, 1H, *J* = 8.5 Hz, 6'-H), 8.51 (dd, 1H, *J* = 8.5 Hz, *J* = 1.5 Hz, 5'-H), 8.72 (s, 1H, 3'-H). ^{13}C-NMR (500 MHz, $CDCl_3$) δ [ppm] = 16.5 (C-18), 24.3, 25.8, 26.6, 29.5, 31.3, 31.5, 35.4, 36.5, 40.6, 42.4, 45.0 (C-13), 48.8, 49.0, 85.7 (C-17), 119.4 (C-3'), 124.6 (C-4), 127.2 (C-5'), 131.5 (C-6'), 132.8 (C-1'), 149.0 (2C: C-2' and C-4'), 163.0 (COO), 166.1 (C-5), 199.7 (C-3).

1.2. 17α-19-nortestosterone-17-yl 3',5'-dinitrobenzoate (7)

Compound **1** (1 mmol) and 530 mg (2.5 mmol) 3,5-dinitrobenzoic acid was treated in 15 ml toulene as described in the general procedure. The resulting crude product was chromatographed on a silica gel column with CH_2Cl_2 yielding pure **7** (230 mg, 47%). Mp: 211–214 °C, $[\alpha]_D^{20}$ – 110 (*c* = 1 in $CHCl_3$). ^{1}H-NMR (500 MHz, $CDCl_3$) δ [ppm] = 0.93 (s, 3H, 18-H_3), 5.20 (d, 1H, *J* = 6.0 Hz, 17-H), 5.84 (s, 1H, 4-H), 9.10 (s, 2H, 2'-H and 6'-H), 9.21 (s, 1H, 4'-H). (Found: C, 64.15; H, 5.97. $C_{25}H_{28}N_2O_7$ (468.50) requires: C, 64.09; H, 6.02 %). ^{13}C-NMR (500 MHz, $CDCl_3$) δ [ppm] = 16.7 (C-18), 24.6, 25.8, 26.6, 29.9, 31.3, 32.0, 35.4, 36.5, 40.6, 42.5, 45.4, (C-13), 49.0, 49.3, 84.8, (C-17), 122.3 (C-4'), 124.8 (C-4), 129.2 (2C: C-2' and C-6'), 134.3 (C-1'), 148.7 (2C: C-3' and C-5'), 162.0 (COO), 165.9 (C-5), 199.6 (C-3).

1.3. 17α-Chloro-19-nortestosterone (3)

Compound **1** (1 mmol) and 208 mg (2.5 mmol) 2-chloropropane was treated in 15 ml toluene as described in general procedure. The resulted crude product was chromatographed on a silicagel column with CH_2Cl_2/hexane (1:3 v,v) yielding pure **3** (247 mg, 79%). Mp: 138–141 °C, [α] – 27 (*c* = 1 in $CHCl_3$). (Found: C, 73.92; H,8.54. $C_{18}H_{25}ClO$ (292.84) requires C, 73.83; H, 8.60 %). ^{1}H-NMR (500 MHz, $CDCl_3$) δ [ppm] = 0.86 (s, 3H, 18-H_3), 4.08 (d, 1H, *J* = 6.0 Hz, 17-H), 5.83 (s, 1H, 4-H). ^{13}C-NMR (500 MHz, $CDCl_3$) δ [ppm] = 18.0 (C-18), 24.3, 26.3, 26.6, 31.3, 33.4, 34.2, 35.5, 40.8, 42.5, 46.2 (C-13), 47.1, 48.9, 70.8 (C-17), 124.6 (C-4), 166.3 (C-5), 199.7 (C-3).

1.4. 17α-Bromo-19-nortestosterone (4)

Compound **1** (1 mmol) and 308 mg (2.5 mmol) 2-bromopropane was treated in 15 ml toluene as described in general procedure. The residual crude product was chromatographed on a silicagel column with CH_2Cl_2/hexane (1:1 v,v) yielding pure **4** (290 mg, 85%). Mp: 146–148 °C , [α] – 38 (*c* = 1 in $CHCl_3$). (Found: C, 64.02; H, 7.38. $C_{18}H_{25}BrO$ (337.29) requires: C, 64.10; H, 7.47 %). ^{1}H-NMR (500 MHz, $CDCl_3$) δ [ppm] = 0.88 (s, 3H, 18-H_3), 4.25 (d, 1H, *J* = 6.5 Hz, 17-H), 5.83 (s, 1H, 4-H). ^{13}C-NMR (500 MHz, $CDCl_3$) δ [ppm] = 17.5 (C-18), 24.5, 26.6, 31.3, 34.8, 35.5, 35.8, 35.9, 36.5, 40.9, 42.5, 46.0 (C-13), 47.3, 48.9, 65.6, (C-17), 124.6 (C-4), 166.3 (C-5), 199.8 (C-3).

1.5. 17α-Iodo-19-nortestosterone (5)

Compound **1** (1 mmol) and 418 mg (2.5 mmol) 2-iodopropane was treated in 15 ml toluene as described in general procedure. The reaction mixture was washed with dilute solution of $Na_2S_2O_3$ (2 x 20 ml). The residue crude product was chromatographed on a silica gel column with CH_2Cl_2/hexane (1:1 v,v) yielding pure **5** (310 mg, 80 %). Mp: 135–137 °C, $[\alpha]_D^{20}$ – 76 (*c* = 1 in $CHCl_3$). ^{1}H-NMR (500 MHz, $CDCl_3$) δ [ppm] = 0.89 (s, 3H, 18-H_3), 4.38 (d, 1H, *J* = 6.5 Hz, 17-H), 5.84 (s, 1H, 4-H). (Found: C, 56.34; H, 6.68. $C_{18}H_{25}IO$ (384.29) requires: C, 56.26; H, 6.56 %). ^{13}C-NMR (500 MHz, $CDCl_3$) δ [ppm] = 15.7 (C-18), 24.7, 26.6, 27.2, 31.3, 35.5, 36.5, 36.7, 40.5, 41.2, 42.5, 45.1 (C-13), 47.5, 47.9, 48.8, (C-17), 124.6 (C-4), 166.2, (C-5), 199.7 (C-3).

1.6. 17α-19-Nortestosterone (2)

Compound **6** (469 mg, 1 mmol) was dissolved in methanol (30 ml) containing $NaOCH_3$ (108 mg, 2 mmol) and the solution was allowed to stand for 24 h. It was then diluted with water, and the white precipitate that separated out was filtered off and crystallized from ethanol/water to obtain **2** (255 mg, 92 %). Mp: 149–151 °C, $[\alpha]_D^{20}$ + 23 (*c* = 1 in $CHCl_3$). (Found: 78.92; H, 9.37. $C_{18}H_{26}O_2$ (274.40) requires: C, 78.79, H, 9.55 %). ^{1}H-NMR (500 MHz, $CDCl_3$) δ [ppm]= 0.73 (s, 3H, 18-H_3), 3.78 (d, 1H, *J* = 5.5 Hz, 17-H), 5.83 (s, 1H, 4-H).

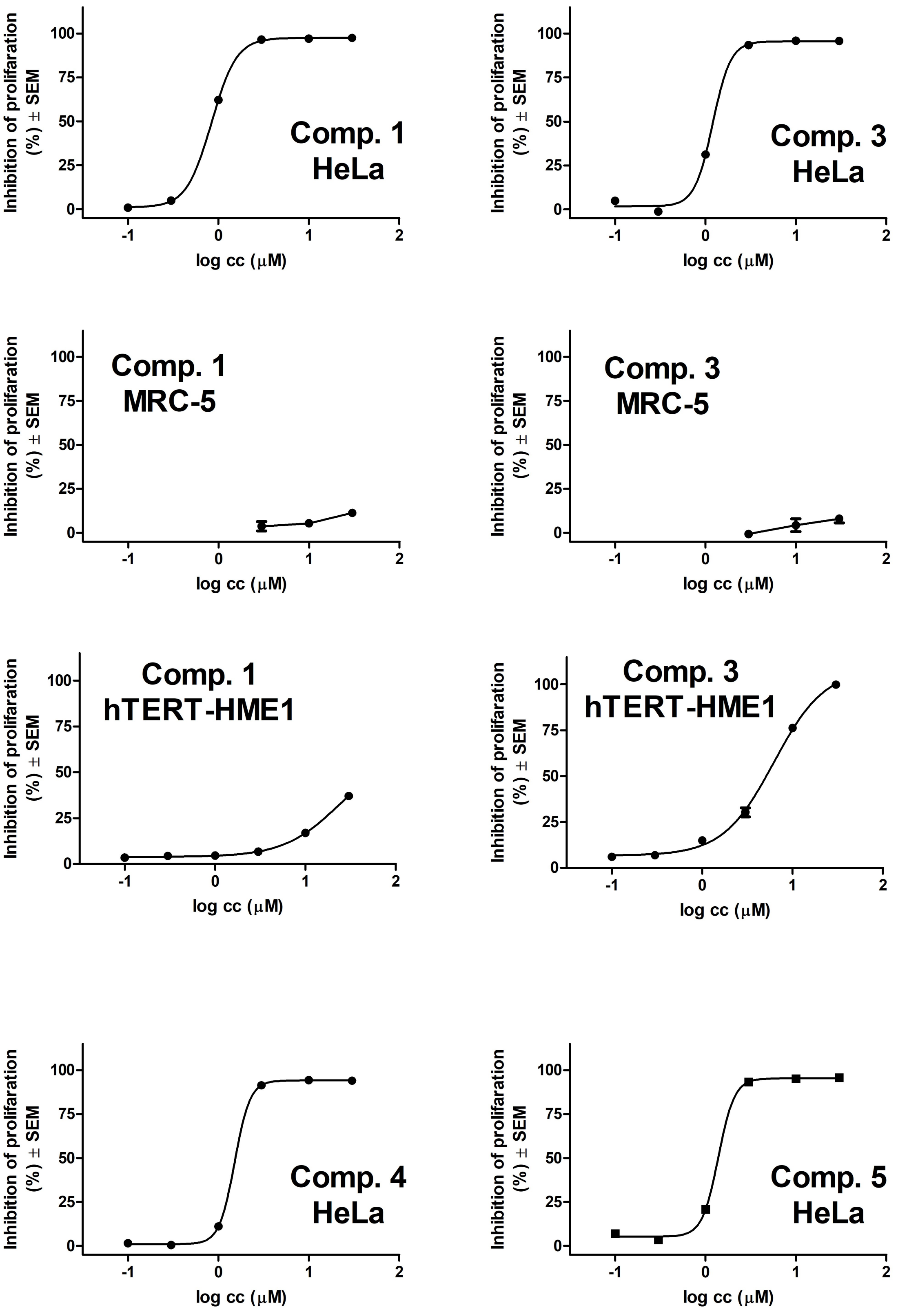

Figure S1. Representative concentration-response curves of the tested compounds.

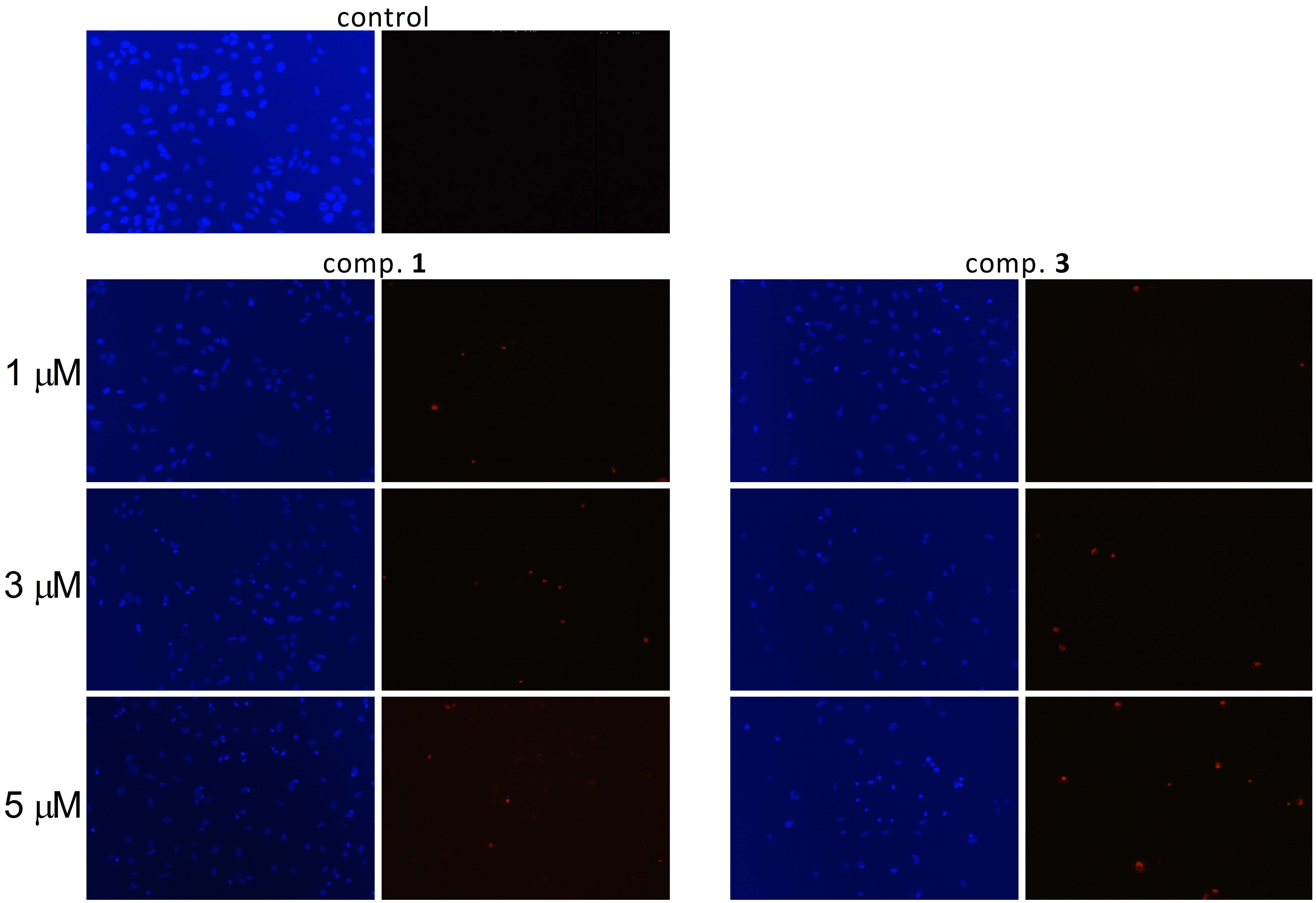

Figure S2. Fluorescence microscopy images of Hoechst 33258-PI staining. Two separate pictures from the same field were taken. The blue fluorescence (left panels) indicates Hoechst 33258 and the red coloration (right panels) indicates propidium accumulation.
